# Sphingosine 1-Phosphate Activation of EGFR As a Novel Target for Meningitic *Escherichia coli* Penetration of the Blood-Brain Barrier

**DOI:** 10.1371/journal.ppat.1005926

**Published:** 2016-10-06

**Authors:** Xiangru Wang, Ravi Maruvada, Andrew J. Morris, Jun O. Liu, Michael J. Wolfgang, Dong Jae Baek, Robert Bittman, Kwang Sik Kim

**Affiliations:** 1 Department of Pediatrics, Division of Pediatric Infectious Diseases, Johns Hopkins University School of Medicine, Baltimore, Maryland, United States of America; 2 State Key Laboratory of Agricultural Microbiology, College of Veterinary Medicine, Huazhong Agricultural University, Wuhan, Hubei, China; 3 Division of Cardiovascular Medicine, The Gill Heart Institute, University of Kentucky, Lexington, Kentucky, United States of America; 4 Department of Pharmacology and Molecular Sciences, Johns Hopkins University School of Medicine, Baltimore, Maryland, United States of America; 5 Department of Biological Chemistry, Center for Metabolism and Obesity Research, Johns Hopkins University School of Medicine, Baltimore, Maryland, United States of America; 6 Department of Chemistry and Biochemistry, Queens College, The City University of New York, Flushing, New York, United States of America; Institut Cochin, FRANCE

## Abstract

Central nervous system (CNS) infection continues to be an important cause of mortality and morbidity, necessitating new approaches for investigating its pathogenesis, prevention and therapy. *Escherichia coli* is the most common Gram-negative bacillary organism causing meningitis, which develops following penetration of the blood–brain barrier (BBB). By chemical library screening, we identified epidermal growth factor receptor (EGFR) as a contributor to *E*. *coli* invasion of the BBB *in vitro*. Here, we obtained the direct evidence that CNS-infecting *E*. *coli* exploited sphingosine 1-phosphate (S1P) for EGFR activation in penetration of the BBB *in vitro* and *in vivo*. We found that S1P was upstream of EGFR and participated in EGFR activation through S1P receptor as well as through S1P-mediated up-regulation of EGFR-related ligand HB-EGF, and blockade of S1P function through targeting sphingosine kinase and S1P receptor inhibited EGFR activation, and also *E*. *coli* invasion of the BBB. We further found that both S1P and EGFR activations occurred in response to the same *E*. *coli* proteins (OmpA, FimH, NlpI), and that S1P and EGFR promoted *E*. *coli* invasion of the BBB by activating the downstream c-Src. These findings indicate that S1P and EGFR represent the novel host targets for meningitic *E*. *coli* penetration of the BBB, and counteracting such targets provide a novel approach for controlling *E*. *coli* meningitis in the era of increasing resistance to conventional antibiotics.

## Author Summary

*Escherichia coli* is the most common Gram-negative bacillary organism causing meningitis, and *E*. *coli* meningitis continues to be an important cause of mortality and morbidity. *E*. *coli* penetration of the blood–brain barrier (BBB) is essential for the development of *E*. *coli* meningitis, but the underlying mechanisms remain incompletely understood. Recent reports of *E*. *coli* strains producing CTX-M-type or TEM-type extended-spectrum β-lactamases, including antimicrobial-resistant *E*. *coli* sequence type 131 (ST131) are of particular concern. These findings necessitate searches for new targets for investigating the pathogenesis and therapeutic development of *E*. *coli* meningitis. Our work demonstrated for the first time that sphingosine 1-phosphate (S1P) activation of epidermal growth factor receptor (EGFR) represents a novel mechanism by which CNS-infecting *E*. *coli* strains penetrate the BBB, and that blockade of S1P and EGFR prevented *E*. *coli* penetration of the BBB. We also determined that the specific *E*. *coli* factors contributing to penetration of the BBB exploit S1P-EGFR signaling, and that c-Src is downstream of S1P-EGFR. Our findings reveal a novel mechanism by which meningitic *E*. *coli* penetrates the BBB, and also demonstrate the novel targets for investigating the pathogenesis, prevention, and therapy of *E*. *coli* meningitis.

## Introduction

Bacterial meningitis is currently recognized as one of the top ten leading causes of global deaths from infectious diseases. Case fatality rates range from 5–25%, and approximately 25–50% of survivors sustain neurologic sequelae [1–4]. The morbidity and mortality rates of bacterial meningitis vary, depending on age, immune state, patient location, and causative organism. Patient groups at risk of high rates of mortality and morbidity include newborns, the elderly, and those living in developing countries, while the infections with higher rates of mortality and morbidity are those caused by Gram-negative bacilli [2,3].

*Escherichia coli* is the most common Gram-negative bacillary organism causing meningitis [1–4]. Most cases of *E*. *coli* meningitis develop from hematogenous spread [5,6], and occur as a result of the bacterial penetration of the blood–brain barrier (BBB), which is a prerequisite for the development of central nervous system (CNS) infection [1–4].

The BBB consists of brain microvascular endothelial cells, astrocytes and pericytes, and is a structural and functional barrier that maintains the neural microenvironment by regulating the passage of molecules into and out of brain, and prevents circulating microbes from penetrating into the brain [1,2]. Meningitis isolates of *E*. *coli*, however, have been shown to invade the BBB [1–4], but it remains incompletely understood how circulating *E*. *coli* strains penetrate the BBB.

Several lines of evidence from human cases and experimental animal models of *E*. *coli* meningitis indicate that *E*. *coli* penetration into the brain follows a high level of bacteremia, and that cerebral capillaries are the portal of entry into the brain [1–6]. Since *E*. *coli* penetration into the brain occurred in the cerebral microvasculature [5], we developed the *in vitro* BBB model with human brain microvascular endothelial cells (HBMEC) to investigate *E*. *coli* invasion of the BBB [7,8]. We also developed the *in vivo* animal model of experimental hematogenous meningitis to mimic *E*. *coli* penetration into the brain that occurs in neonatal meningitis [5]. We have shown with both *in vitro* and *in vivo* models that *E*. *coli* invasion of HBMEC is directly correlated with its penetration into the brain [9–15], suggesting that elucidation of the mechanisms involved in *E*. *coli* invasion of HBMEC is likely to enhance our knowledge on the pathogenesis of *E*. *coli* meningitis.

We took advantage of genome sequencing information available from meningitis isolates of *E*. *coli* (e.g., strains IHE3034, S88, RS218) to study *E*. *coli* penetration of the BBB. Using functional genomics studies (e.g., transposon and signature-tagged mutagenesis, DNA microarray and comparative genome hybridization), we have identified several microbial factors contributing to meningitic *E*. *coli* invasion of HBMEC, which include OmpA, FimH, NlpI, IbeA, IbeB, IbeC and CNF1 [9–12,15–22]. We have also shown that these microbial factors exploit specific host receptors and host cell signaling molecules for bacterial invasion of HBMEC [2,3]. For example, OmpA interacts with gp96 on HBMEC, resulting in activation of focal adhesion kinase (FAK), while FimH interaction with CD48 and CNF1 interaction with 37 kDa laminin receptor precursor (37LRP) lead to activation of RhoGTPases [23–27]. Biological relevance of these microbial-host interactions in the pathogenesis of *E*. *coli* meningitis is shown by the demonstrations that (a) exogenous OmpA and gp96 and anti-gp96 antibodies block *E*. *coli* invasion of HBMEC, but do not exhibit any blocking effect on the OmpA mutant [23,24], (b) addition of exogenous FimH- or CD48- antibodies inhibits *E*. *coli* invasion of the HBMEC [25], and (c) expression levels of 37LRP dictates the ability of *E*. *coli* to invade HBMEC, but exhibited no effect on the CNF1 mutant [26,27]. Despite the extensive information available on microbial and host factors as well as host cell signaling molecules contributing to *E*. *coli* invasion of HBMEC [2–4], the mechanisms involved in *E*. *coli* penetration of the BBB remain incompletely understood.

Since meningitic *E*. *coli* invasion of HBMEC is correlated with its penetration into the brain, we used *E*. *coli* invasion of HBMEC as a biologically relevant model in the present study for screening of a chemical library to discover novel targets affecting *E*. *coli* penetration of the BBB. In our screen, we identified that gefitinib, a selective inhibitor of epidermal growth factor receptor (EGFR) tyrosine kinase [28], significantly inhibited *E*. *coli* invasion into HBMEC monolayers. EGFR belongs to the ErbB family of receptor tyrosine kinases (RTKs), consisting of four closely-related members (ErbB1/EGFR, ErbB2, ErbB3, ErbB4) [29–31]. EGFR is initially expressed in the plasma membrane in an inactive form, and becomes activated through certain kinases and/or after binding to its specific ligands, which are produced as transmembrane precursors and released by proteolytic cleavage [29–32]. To date, several bacterial pathogens have been reported to target EGFR through different mechanisms to facilitate their infection of host cells, including *Neisseria gonorrhoeae*, *Neisseria meningitidis*, *Helicobacter pylori*, *Haemophilus influenzae*, and *Klebsiella pneumoniae* [33–38]. However, to our knowledge, it is unknown whether EGFR is involved in meningitic *E*. *coli* invasion of the BBB. In the present study, we reported for the first time that sphingosine 1-phosphate (S1P)-mediated activation of EGFR represents a novel mechanism exploited by meningitic *E*. *coli* for penetration of the BBB, the essential step in the development of *E*. *coli* meningitis. EGFR as well as S1P are, therefore, likely to represent the novel targets for investigating the pathogenesis, prevention and therapy of *E*. *coli* meningitis.

## Results

### Meningitic *E*. *coli* exploits EGFR for its penetration of the BBB

**Fig 1:**
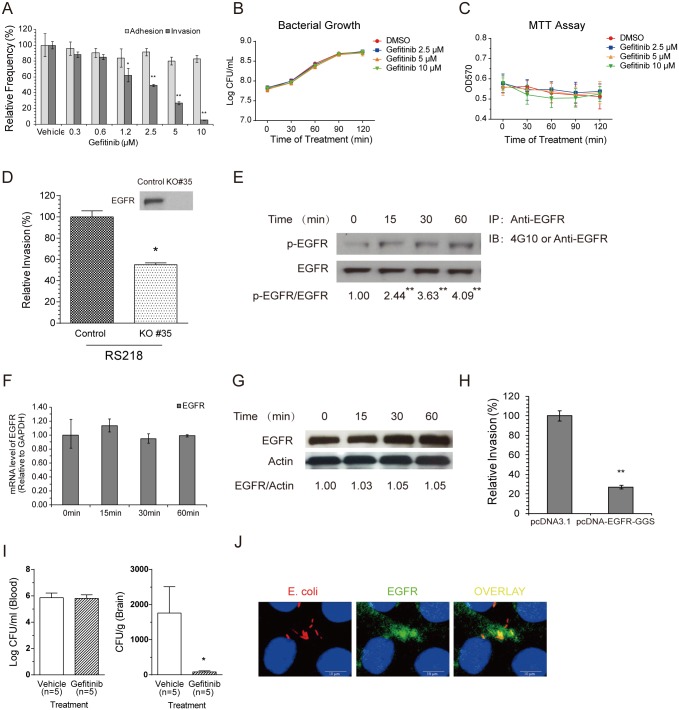
Meningitic *E*. *coli* exploits EGFR for its penetration of the BBB *in vitro* and *in vivo*. (A) The EGFR-selective inhibitor gefitinib inhibits meningitic *E*. *coli* RS218 invasion of HBMEC in a dose-dependent manner, but does not affect its adhesion. * *p*<0.05, ** *p*<0.01. (B) Bacterial growth was not affected by the treatment with gefitinib. Overnight bacterial culture was 1:100 transferred into fresh medium with or without gefitinib at indicated concentrations, and further incubated for 2 h. Viable bacterial counts were determined by series dilution and plating at 30 min interval. (C) Gefitinib did not lead to an inhibition of cell proliferation when used at the indicated concentrations. (D) Meningitic *E*. *coli* invasion of the EGFR knock-out HBMEC (KO#35) was significantly decreased compared to the invasion of the control cells. * *p*<0.05. (E) A time-dependent activation of EGFR occurs in response to *E*. *coli* RS218 in HBMEC. The ratio of p-EGFR and EGFR was calculated based on densitometry analysis. ** indicates the difference was significant compared to time 0 (*p*<0.01). (F) EGFR mRNA transcription levels did not change in response to *E*. *coli* RS218 in HBMEC, as assessed by real-time PCR analysis. GAPDH was used as the endogenous reference. Representative results from three individual experiments are shown. (G) EGFR protein expression levels were not affected in response to *E*. *coli* RS218 in HBMEC. Actin was probed in the same lysate and used as a loading control. (H) RS218 invasion was significantly reduced in HBMEC transfected with the dominant-negative EGFR construct pcDNA-EGFR-GGS compared with pcDNA3.1 control vector-transfected HBMEC. ** *p*<0.01. (I) *E*. *coli* RS218 penetration into the brain was significantly inhibited by administration of gefitinib (10 mg/kg) compared with vehicle treatment. In contrast, the magnitudes of bacteremia did not differ between the recipients of gefitinib and vehicle control. * *p* <0.05. (J) Co-localization of *E*. *coli* strain RS218 and EGFR is demonstrated in HBMEC. Scale bar = 10 μm.

Our chemical screen identified gefitinib as an inhibitor of meningitic *E*. *coli* invasion of HBMEC monolayer. Gefitinib is a low-molecular-weight anilinoquinazoline that selectively inhibits EGFR [28]. EGFR has been shown to play essential roles in cell proliferation, survival, and migration as well as in carcinogenesis and cancer progression, and is considered an attractive target for anticancer therapies [29–31]. However, the role of EGFR in *E*. *coli* meningitis is unknown. To address this issue, we first determined the effect of gefitinib on meningitic *E*. *coli* strain RS218 binding to and invasion of HBMEC. We found that gefitinib inhibited RS218 invasion of HBMEC in a dose-dependent manner without affecting *E*. *coli* adhesion to HBMEC (Fig 1A), suggesting that its target, EGFR, is likely to be involved in *E*. *coli* invasion of the BBB. Gefitinib did not affect bacterial growth, as assessed by determination of colony-forming units (CFUs) in the presence and absence of gefitinib (Fig 1B). The cytotoxicity and proliferation of the HBMEC, as assessed by live/dead stain (Molecular Probes) and MTT assays, were not affected by gefitinib (Fig 1C). EGFR was subsequently knocked out from HBMEC via CRISPR-Cas9 editing approach, and bacterial invasion of the EGFR knock-out cells (KO#35) were compared with that of the control cells. The EGFR was not detectable in the KO#35 HBMEC by the Western blotting (Fig 1D), and RS218 invasion of the KO#35 cells was significantly decreased compared with that of the control cells (Fig 1D). We next examined the contribution of EGFR tyrosine kinase activity to *E*. *coli* invasion of HBMEC. We showed a time-dependent tyrosine phosphorylation of EGFR in response to meningitic *E*. *coli* RS218 infection (Fig 1E) but no change in EGFR transcription or expression (Fig 1F and 1G). *E*. *coli* invasion of HBMEC transfected with dominant-negative EGFR, pcDNA-EGFR-GGS, encoding EGFR without tyrosine kinase activity [39], was significantly decreased compared with that of control vector-transfected cells (Fig 1H). Moreover, we examined the role of EGFR in meningitic *E*. *coli* penetration into the brain in a neonatal animal model, involving intraperitoneal administration of gefitinib to 1-week-old mice. The results, as determined by bacterial counts (CFUs) recovered from the blood and brain specimens of mice receiving gefitinib or vehicle control, showed that gefitinib did not affect the level of bacteremia, but was efficacious in preventing *E*. *coli* penetration into the brain (Fig 1I). In addition, we showed a co-localization of EGFR with meningitic *E*. *coli* strain RS218 in HBMEC monolayer (Fig 1J). Together, these data support our novel concept that EGFR contributes to meningitis-causing *E*. *coli* penetration of the BBB.

**Fig 2:**
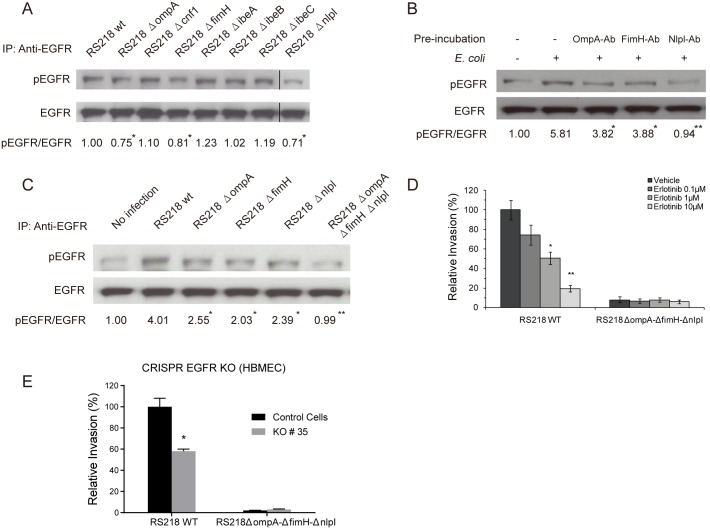
OmpA, FimH, and NlpI proteins are involved in meningitic *E*. *coli*-induced activation of EGFR. (A) *E*. *coli* mutants with deletion of *ompA*, *fimH* or *nlpI* exhibited lower EGFR activation compared with wild-type RS218 in HBMEC monolayer. * *p*<0.05 compared to wild type bacteria. (B) Antibodies directed against OmpA, FimH, and NlpI decreased EGFR activation in response to *E*. *coli* in HBMEC. The bacteria were preincubated with the antibodies (with 1:10 dilution) individually for 1 h, and then added to HBMEC and incubated for 30 min for assessment of EGFR activation. * *p*<0.05, ** *p*<0.01 compared to *E*. *coli* infection without antibody incubation. (C) EGFR activation in response to *E*. *coli* was not discernible with the triple deletion mutant (RS218*ΔompAΔfimHΔnlpI*), similar to that of the uninfected control HBMEC. * *p*<0.05, ** *p*<0.01 compared with the *E*. *coli* RS218 wild-type infection. (D) Erlotinib inhibited the wild-type strain RS218 invasion of HBMEC in a dose-dependent manner, while it did not affect the HBMEC invasion by the triple mutant strain with deletion of *ompA*, *fimH*, and *nlpI*. * *p*<0.05, ** *p*<0.01. (E) *E*. *coli* wild-type strain RS218 invasion was significantly decreased in EGFR knock-out HBMEC (KO#35 cells) (* *p*<0.05), while the triple deletion mutant’s invasion did not differ between knock-out and control cells.

As indicated above, we demonstrated both *in vitro* and *in vivo* that EGFR is involved in meningitic *E*. *coli* RS218 penetration of the BBB, but there was no information on how EGFR is activated in response to *E*. *coli* invasion. Since *E*. *coli* penetration of the BBB requires specific microbial factors that contribute to HBMEC invasion [1–4], we examined whether EGFR activation occurred in response to those *E*. *coli* factors contributing to HBMEC invasion. The wild-type strain RS218 and its mutants with deletion of *ompA*, *cnf1*, *fimH*, *ibeA*, *ibeB*, *ibeC* or *nlpI* were examined for their involvement in EGFR tyrosine phosphorylation in HBMEC. We found that the mutants deleted of *ompA*, *fimH* or *nlpI* exhibited a significantly lower level of EGFR activation compared with wild-type strain RS218 (Fig 2A), suggesting that *E*. *coli* OmpA, FimH, and NlpI proteins are likely to contribute to EGFR activation in HBMEC. In contrast, the mutants deleted of *ibeA*, *ibeB*, *ibeC* or *cnf1* did not show the decrease in EGFR activation (Fig 2A). The involvement of OmpA, FimH and NlpI in EGFR activation was further supported by the demonstration that antibodies directed against OmpA, FimH and NlpI inhibited EGFR activation in response to strain RS218 in HBMEC (Fig 2B). As expected, the triple mutant deleted of *ompA*, *fimH* and *nlpI* could not induce a discernible EGFR activation, similar to that of uninfected HBMEC (Fig 2C). The exploitation of EGFR by OmpA, FimH and NlpI in *E*. *coli* invasion of the cells was further examined by using another selective EGFR inhibitor, erlotinib [40], as well as the EGFR KO#35 cells. The results showed that erlotinib inhibited invasion of the wild-type strain RS218 in a dose-dependent manner, while it did not affect the HBMEC invasion by the triple mutant deleted of *ompA*, *fimH*, and *nlpI* (Fig 2D). Similarly, the triple mutant’s invasion of the KO#35 cells did not differ from that of the control cells (Fig 2E). Taken together, these findings indicate that *E*. *coli* virulence factors OmpA, FimH, and NlpI are likely to be involved in exploitation of EGFR in meningitic *E*. *coli* invasion of HBMEC.

### SphK2-S1P-S1P_2_ signaling cascade contributes to *E*. *coli* penetration of the BBB

**Fig 3:**
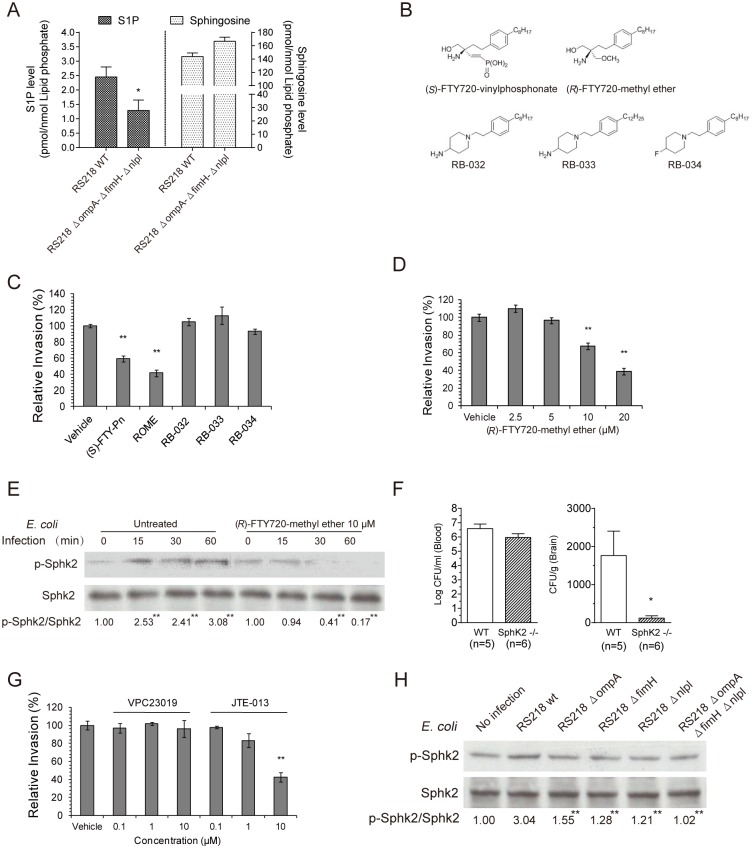
SphK2-S1P-S1P_2_ mediates meningitic *E*. *coli* penetration of the BBB *in vitro* and *in vivo*. (A) S1P generation was significantly higher in HBMEC incubated with wild-type RS218 compared with the triple mutant deleted of *ompA*, *fimH* and *nlpI*. Correspondingly, the sphingosine level was lower in HBMEC incubated with wild-type RS218 than those incubated with the triple deletion mutant. * *p*<0.05. (B) Structures of (*S*)-FTY720-vinylphosphonae (SphK1 and SphK2 inhibitor), (*R*)-FTY720-methyl ether (selective SphK2 inhibitor), RB-032 and RB-033 (selective SphK1 inhibitors), and RB-034 (inactive analogue). (C) Both (*S*)-FTY720-vinylphosphonae (SphK1 and SphK2 inhibitor, shown as (*S*)-FTY-Pn) and (*R*)-FTY720-methyl ether (SphK2 inhibitor, shown as ROME) significantly inhibited RS218 invasion of HBMEC, while the SphK1 inhibitors (RB-032 and RB-033) and inactive analogue (RB-034) did not exhibit any inhibition. ** *p*<0.01. The inhibitors were all used at 10 μM. (D) (*R*)-FTY720-methyl ether inhibited *E*. *coli* RS218 invasion of HBMEC in a dose-dependent manner. ** *p*<0.01. (E) *E*. *coli* RS218 activated SphK2 in a time-dependent manner in HBMEC, while such activation was abolished by pretreatment with 10 μM (*R*)-FTY720-methyl ether. ** *p*<0.01. (F) *E*. *coli* penetration into the brain was significantly less in SphK2 ^−/−^ mice compared with wild-type mice. In contrast, the levels of bacteremia did not differ between the two groups of mice. (G) JTE-013 (S1P_2_ antagonist) significantly inhibited *E*. *coli* invasion of HBMEC, while VPC23019 (S1P_1_ and S1P_3_ antagonist) did not exhibit any inhibition. ** *p*<0.01. (H) The mutants with deletion of *ompA*, *fimH*, or *nlpI* as well as the triple mutant (*ΔompAΔfimHΔnlpI*) induced significantly lower levels of SphK2 activation in HBMEC, compared with wild-type RS218. ** *p*<0.01.

Recent studies have shown that S1P acts as a multifunctional bioactive sphingolipid metabolite implicated in a wide range of biological effects, such as cell proliferation, immune and allergic reactions, and regulation of the vascular cell function [41–44]. The role of S1P in *E*. *coli* meningitis, however, has not been previously appreciated. In our study, we found that the same microbial factors involved in EGFR activation also contributed to S1P generation in response to *E*. *coli* infection in HBMEC. S1P levels were significantly higher in HBMEC infected with meningitic *E*. *coli* wild-type strain RS218 compared to those infected with the triple mutant deleted of *ompA*, *fimH* and *nlpI*, as measured by LC-MS/MS after lipid extraction of HBMEC [45]. The S1P content in HBMEC was normalized to lipid phosphate in the extracted samples and expressed as pmol/nmol lipid phosphate (mean ± SD of three samples), being 2.45 ± 0.35 after 30 min at 37°C in HBMEC incubated with wild-type RS218 vs. 1.29 ± 0.36 in cells incubated with the triple deletion mutant (Fig 3A, *p*<0.05). Accordingly, a trend for the decreased amount of sphingosine was observed in HBMEC incubated with wild-type RS218 compared to that incubated with the triple mutant (Fig 3A). Since the same microbial factors contributing to HBMEC invasion are involved in EGFR activation and S1P generation, we hypothesized that the contribution of EGFR to *E*. *coli* penetration of the BBB is likely to be related to that of S1P.

S1P synthesis is catalyzed by sphingosine kinases 1 and 2 (SphK1 and SphK2) [41–44]. We, therefore, examined the role of S1P in *E*. *coli* invasion of the BBB by determining the involvement of SphK1 and SphK2 using specific inhibitors against SphK1 and/or SphK2, and an inactive analogue (Fig 3B) [46–50]. Both (*S*)-FTY720-vinylphosphonate (inhibitor of SphK1 and SphK2, abbreviated as (*S*)-FTY-Pn) and (*R*)-FTY720-methyl ether (selective inhibitor of SphK2, abbreviated as ROME) significantly inhibited *E*. *coli* RS218 invasion of HBMEC, while SphK1 inhibitors (RB-032 and RB-033) and the inactive analogue (RB-034) did not exhibit any inhibition (Fig 3C), suggesting the involvement of SphK2, not SphK1, in meningitic *E*. *coli* invasion of HBMEC. This finding was further supported by the dose-dependent inhibition of *E*. *coli* RS218 invasion by the SphK2 inhibitor (Fig 3D), as well as the time-dependent activation of SphK2 (analyzed as the ratio of p-SphK2/SphK2) in response to strain RS218 in HBMEC, which was abolished in HBMEC pretreated with the SphK2 inhibitor (Fig 3E). Next, the role of SphK2 in *E*. *coli* penetration into the brain was examined in SphK2^−/−^ mice compared with wild-type C57BL/6j mice [42]. We found that the magnitudes of bacteremia did not differ between the two groups of mice, as shown by the similar bacterial counts in the blood of wild-type and SphK2^−/−^ mice (Fig 3F). However, the bacterial counts in the brains of SphK2^−/−^ mice were significantly lower than those in the brains of wild-type mice (Fig 3F), indicating that deletion of SphK2 resulted in decreased *E*. *coli* penetration into the brain without affecting the magnitude of bacteremia. These *in vitro* and *in vivo* findings demonstrate the novel concept that SphK2 contributes to meningitic *E*. *coli* penetration of the BBB.

S1P is known to exhibit diverse activities by binding to and signaling through its specific cell-surface receptors, which are members of the G protein-coupled receptors (GPCR) family [41,43,44]. We next determined the role of S1P receptors in meningitic *E*. *coli* invasion of HBMEC using selective receptor antagonists (VPC23019 for S1P_1_ and S1P_3_, and JTE-013 for S1P_2_) [41,44]. We found that pretreatment of HBMEC with VPC23019 at indicated concentrations had no effect on *E*. *coli* invasion (Fig 3G), suggesting that receptors S1P_1_ and S1P_3_ are not likely to be involved in *E*. *coli* invasion of the BBB. In contrast, HBMEC pretreated with S1P_2_-specific antagonist JTE-013 displayed a dose-dependent decrease in *E*. *coli* invasion (Fig 3G), suggesting that S1P_2_ plays a role in meningitic *E*. *coli* invasion of HBMEC. Taken together, our findings thus far support the concept that meningitic *E*. *coli* infection of HBMEC increases the generation of S1P through SphK2 activation, and that the interaction of S1P with S1P_2_ is involved in *E*. *coli* invasion of the BBB.

As indicated above, we showed that S1P levels were significantly less in HBMEC incubated with the triple mutant deleted of *ompA*, *fimH* and *nlpI*, compared to wild type strain. To further support our hypothesis that the contribution of EGFR to *E*. *coli* penetration of the BBB is related to that of S1P, the *E*. *coli* factors involved in EGFR activation (OmpA, FimH, NlpI) were examined for their contributions to SphK2 activation in HBMEC. The results revealed that phosphorylation of SphK2 was decreased in HBMEC infected with the mutants with deletion of *ompA*, *fimH* and *nlpI*, individually and in combination, compared with HBMEC infected with wild-type RS218 (Fig 3H). Therefore, these findings support that *E*. *coli* factors OmpA, FimH and NlpI, the key contributors to bacterial adhesion and invasion, participate in both EGFR activation and SphK2-S1P-S1P2 signaling cascade, and suggest a linkage between EGFR and SphK2-S1P-S1P_2_ in meningitic *E*. *coli* penetration of the BBB.

### Meningitic *E*. *coli*-induced activation of EGFR requires SphK2-S1P-S1P_2_ signaling and SphK2-S1P-S1P_2_-mediated upregulation of HB-EGF in HBMEC

**Fig 4:**
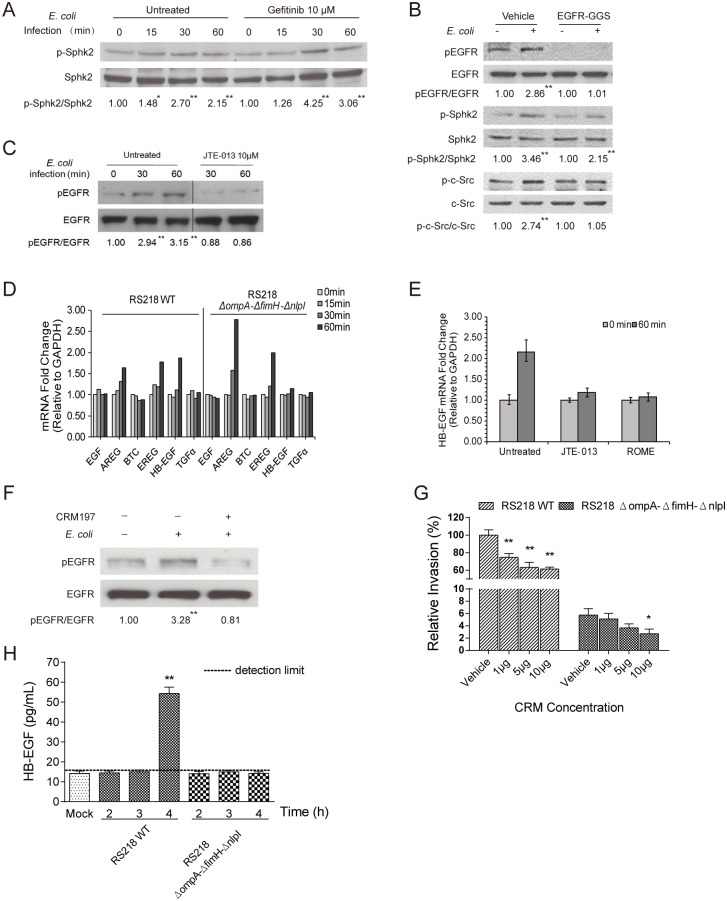
SphK2-S1P-S1P_2_ is upstream of EGFR activation in meningitic *E*. *coli* invasion of HBMEC and contributes to HB-EGF mediated transactivation of EGFR. (A) Activation of SphK2 in response to RS218 did not differ between HBMEC with and without gefitinib pretreatment. * *p*<0.05, ** *p*<0.01. (B) SphK2 activation was not affected in HBMEC expressing dominant-negative EGFR, while EGFR activation was, as expected, abolished in HBMEC expressing dominant-negative EGFR. Activation of c-Src occurred in response to *E*. *coli* in vector-transfected HBMEC, but did not occur in HBMEC expressing dominant-negative EGFR. ** *p*<0.01. (C) JTE-013 (S1P_2_ antagonist) inhibited EGFR activation in response to *E*. *coli* in HBMEC. ** *p*<0.01. (D) Real-time PCR analysis of the expression of EGFR ligands in response to wild-type *E*. *coli* RS218 or the triple deletion mutant in HBMEC. Representative results from three independent assays are shown. GAPDH was used as an endogenous reference. (E) Pretreatment of HBMEC with JTE-013 or (*R*)-FTY720-methyl ether (shown as ROME) prevented HB-EGF up-regulation (analyzed by real-time PCR) in response to RS218. (F) Pretreatment of HBMEC with CRM197 prevented EGFR activation in response to RS218. ** *p*<0.01. (G) CRM197 dose-dependently inhibited RS218 invasion of HBMEC, while only the highest dosage of CRM197 significantly affected HBMEC invasion by the triple mutant. ** *p*<0.01. (H) The release of HB-EGF from HBMEC infected with the triple deletion mutant for up to 4 h was below the detection limit, while HB-EGF release was significantly increased by approximately 3-fold from the cells infected with wild-type RS218 at 4 h, ** *p*<0.01.

Since the same microbial factors (OmpA, FimH, NlpI) are involved in SphK2 activation, S1P generation, and EGFR activation, we examined the potential relationship between the SphK2-S1P-S1P_2_ cascade and EGFR activation in meningitic *E*. *coli* invasion of the BBB. We found that pharmacological inhibition of EGFR with gefitinib did not affect *E*. *coli*-induced phosphorylation of SphK2, as shown by the time-dependent activation of SphK2 in response to meningitic *E*. *coli* in HBMEC with and without gefitinib treatment (Fig 4A). We next compared *E*. *coli*-induced SphK2 phosphorylation in HBMEC transfected with EGFR dominant-negative construct pcDNA-EGFR-GGS with that in cells transfected with the vehicle control pcDNA3.1. The results showed that the pattern of increased SphK2 phosphorylation upon infection with meningitic *E*. *coli* was similar between HBMEC expressing dominant-negative EGFR and HBMEC expressing the control vector (Fig 4B). These findings indicate that pharmacological inhibition and dominant-negative expression of EGFR did not interfere with SphK2 activation by meningitic *E*. *coli*. Next, we examined and compared the EGFR activation upon *E*. *coli* infection in HBMEC, with and without inhibition of S1P function. As shown in Fig 4C, blockade of the S1P signaling cascade by the S1P_2_ antagonist JTE-013 was effective in inhibiting EGFR activation in response to meningitic *E*. *coli*. These findings demonstrate the novel concept that both SphK2-S1P-S1P_2_ and EGFR contribute to meningitic *E*. *coli* invasion, and that the SphK2-S1P-S1P_2_ signaling cascade is likely to act upstream of EGFR in meningitic *E*. *coli* penetration of the BBB.

EGFR consists of an extracellular ligand-binding domain, a single membrane-spanning region, and a cytoplasmic kinase domain [31,51,52]. It is known that EGFR activation occurs via kinases and/or transactivation through binding to specific ligands. At present, several EGFR-related ligands are known, including EGF, transforming growth factor α (TGFα), heparin-binding EGF-like ligand (HB-EGF), amphirugulin (AREG), betacellulin (BTC), epiregulin (EREG), epigen and neuregulin family members [31,51,52]. We next performed quantitative real-time PCR to investigate whether expressions of the above-mentioned ligands are affected in response to meningitic *E*. *coli*. We selected six ligands, EGF, AREG, BTC, EREG, HB-EGF, and TGFα, representing the EGFR-related ligands previously examined in a study with *N*. *gonorrhoeae*, and determined their transcriptional levels in response to meningitic *E*. *coli* infection. Our quantitative PCR data showed that three ligands, HB-EGF, AREG and EREG, displayed significant up-regulation at 60 min after infection with strain RS218, while the transcriptional levels of EGF, BTC and TGFα remained unchanged during the infection (Fig 4D). Since the *E*. *coli* factors OmpA, FimH, and NlpI were shown to be important in EGFR activation, we examined whether the transcriptional levels of those ligands were changed in response to the triple deletion mutant (*ΔompAΔfimHΔnlpI*), compared with the wild-type strain. The results showed that the triple deletion mutant was able to induce significant up-regulation of AREG and EREG after 60 min, while there was no up-regulation of HB-EGF, as well as EGF, BTC, and TGFα, after infection with the triple deletion mutant (Fig 4D). These findings demonstrate that HB-EGF is the ligand up-regulated in response to meningitic *E*. *coli* strain RS218, compared with the triple mutant with deletion of *ompA*, *fimH*, and *nlpI*, suggesting that increased expression of HB-EGF might be involved in the increased activation of EGFR by meningitic *E*. *coli* strain. As shown above, the SphK2-S1P-S1P_2_ signaling cascade is shown to be upstream of EGFR, and we determined the effect of SphK2-S1P-S1P_2_ blockade on up-regulation of HB-EGF in response to *E*. *coli*. As expected, we observed that HB-EGF up-regulation in response to strain RS218 was abolished in HBMEC pretreated with SphK2 inhibitor ROME and S1P_2_ antagonist JTE-013 (Fig 4E). Taken together, these findings support that the SphK2-S1P-S1P_2_ cascade exploits EGFR activation via up-regulation of HB-EGF in response to meningitic *E*. *coli*, and that SphK2-S1P-S1P_2_ is upstream of EGFR in meningitic *E*. *coli* invasion of HBMEC.

HB-EGF is synthesized as a membrane-spanning precursor molecule and proteolytically processed by metalloproteinases of the ADAM (a disintegrin and metalloproteinase) family to be involved in binding to and activation of EGFR [53,54]. To further examine the contribution of HB-EGF to EGFR activation and *E*. *coli* invasion of the BBB, we investigated the effect of HB-EGF shedding on EGFR activation and *E*. *coli* invasion of HBMEC using the diphtheria toxin mutant Cross-Reacting Material 197 (CRM197), a nontoxic mutant of the diphtheria toxin that retains the ability to bind pro-HB-EGF and prevent its shedding from EGFR stimulation [55]. The results showed that CRM197 was effective in preventing EGFR activation in response to *E*. *coli* strain RS218 (Fig 4F), and also significantly inhibited RS218 invasion of HBMEC in a dose-dependent manner. The triple deletion mutant, as expected, exhibited significantly decreased HBMEC invasion and CRM197 did not decrease the triple mutant’s invasion, and significant inhibition was demonstrated at the highest concentration tested (Fig 4G). As shown previously [1–4], HBMEC invasion frequency of less than 5% of wild type strain’s invasion, however, is less likely to be biologically relevant. We next examined and compared the release of secretory HB-EGF in the supernatants of HBMEC in response to infection with wild-type strain RS218 or its triple deletion mutant by ELISA. The HB-EGF levels in HBMEC infected with the triple deletion mutant for up to 4 h were below the detection limit (16 pg/mL), similar to those of the uninfected control. In contrast, the HB-EGF levels in HBMEC infected with wild-type RS218 were increased by approximately 3-fold at 4 h of infection (*p*<0.01) (Fig 4H).

Taken together, the above findings demonstrate that meningitic *E*. *coli* RS218, with the help of specific microbial factors OmpA, FimH and NlpI, up-regulates the expression and release of HB-EGF, resulting in the transactivation of EGFR, and that this transactivation is dependent on the SphK2-S1P-S1P_2_ signaling cascade.

### c-Src tyrosine kinase participates in *E*. *coli* invasion of HBMEC and is downstream of the S1P-EGFR pathway

**Fig 5:**
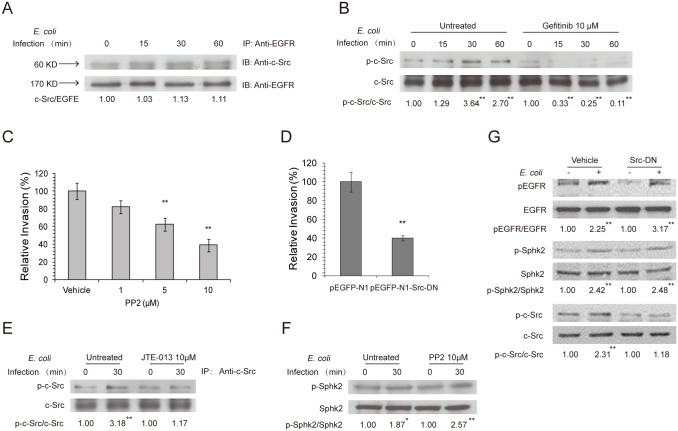
S1P and EGFR promote meningitic *E*. *coli* invasion of HBMEC monolayer *via* exploiting c-Src. (A) Association of c-Src with EGFR in response to *E*. *coli* in HBMEC, as shown by co-immunoprecipitation of HBMEC lysates with an anti-EGFR antibody. (B) c-Src activation occurred in response to *E*. *coli* in a time-dependent manner in HBMEC, but was abolished by pretreatment with gefitinib. (C) Pretreatment of HBMEC with PP2 (Src inhibitor) exhibited a dose-dependent inhibition of *E*. *coli* RS218 invasion. ** *p*<0.01. (D) *E*. *coli* RS218 invasion was significantly reduced in HBMEC expressing the dominant-negative Src construct, pEGFP-N1-Src-DN, compared with the vector (pEGFP-N1)-transfected HBMEC. ** *p*<0.01. (E, F) Pretreatment of HBMEC with JTE-013 (S1P_2_ antagonist) inhibited c-Src activation in response to *E*. *coli* (E), while pretreatment with PP2 (Src inhibitor) did not affect SphK2 activation (F). * *p*<0.05, ** *p*<0.01. (G) *E*. *coli* activation of EGFR and SphK2 was not affected in HBMEC expressing dominant-negative c-Src, while c-Src activation was, as expected, abolished in HBMEC transfected with dominant-negative c-Src compared with vector control-transfected HBMEC. ** *p*<0.01.

Meningitic *E*. *coli* strains exploit specific host cell signaling molecules to promote their invasion of the BBB [1–4]. The phosphotyrosine residues in the cytoplasmic domain of EGFR can serve as bait for recruitment of proteins containing SH2 domains or certain phosphotyrosine-binding domains, depending on stimuli, and can act as a switch to assist and extend the signal transduction of RTK pathways [29]. The c-Src tyrosine kinase was shown to be recruited by the phosphotyrosine residues of EGFR upon activation and to function as a mediator of EGFR signaling, e.g., ligand-independent activation of EGFR [56]. Moreover, c-Src tyrosine kinase was reported to regulate host cell actin cytoskeleton rearrangement and contribute to *E*. *coli* invasion of HBMEC [57]. To examine whether c-Src tyrosine kinase is involved in EGFR signaling in response to meningitic *E*. *coli* invasion, we performed co-immunoprecipitation and Western blotting to assess the possible recruitment and activation of c-Src by EGFR. Our co-immunoprecipitation experiments with an anti-EGFR antibody showed that c-Src tyrosine kinase (60 kD) was associated with EGFR and that this association was maintained in HBMEC during 60 min incubation with meningitic *E*. *coli* RS218 (Fig 5A). After stripping, the membrane was re-probed for EGFR, showing an unchanged level of EGFR upon *E*. *coli* invasion (Fig 5A), consistent with our earlier demonstration of no change in EGFR expression in response to meningitic *E*. *coli* infection. We subsequently examined the activation of c-Src tyrosine kinase in response to *E*. *coli* RS218 infection, and showed that c-Src activation occurred in a time-dependent manner, but was abolished in HBMEC pretreated with the EGFR kinase inhibitor gefitinib (Fig 5B) as well as in HBMEC expressing the EGFR dominant-negative construct (Fig 4B). These findings suggest that c-Src tyrosine kinase acts downstream of EGFR in meningitic *E*. *coli* invasion of HBMEC.

We next examined the contribution of c-Src to *E*. *coli* invasion of HBMEC. As expected from the c-Src association with EGFR, *E*. *coli* invasion of HBMEC was significantly decreased by pretreatment of HBMEC with the c-Src tyrosine kinase inhibitor PP2 compared with the vehicle control, as well as by transfection of HBMEC with c-Src dominant-negative construct compared with the control vector (Fig 5C and 5D). In support of our demonstration that c-Src is downstream of EGFR activation, which is in turn a downstream event of SphK2-S1P-S1P_2_ signaling in *E*. *coli* invasion of HBMEC, we found that pretreatment of HBMEC with the S1P_2_ antagonist JTE-013 inhibited c-Src activation in response to *E*. *coli* RS218 (Fig 5E), while HBMEC pretreated with the c-Src kinase inhibitor PP2 or transfected with the c-Src dominant-negative construct did not affect SphK2 and EGFR activation in response to RS218 infection in HBMEC (Fig 5F and 5G). Taken together, these findings demonstrate that c-Src tyrosine kinase is downstream of the SphK2-S1P-S1P_2_-EGFR signaling cascade and that SphK2-S1P-S1P_2_-EGFR-c-Src contribute to meningitic *E*. *coli* invasion of the BBB.

## Discussion

Bacterial pathogens, including meningitis-causing pathogens, exploit host cell signaling molecules to promote their infections, but the underlying mechanisms vary depending upon the types of pathogens and host tissues. Recent studies have shown that EGFR plays a role for several pathogenic organisms in the pathogenesis of their infections. For example, *Pseudomonas aeruginosa* and *H*. *pylori* were shown to induce transactivation of EGFR to prevent epithelial cell apoptosis during the early stage of infection, thereby facilitating their survival in the host cells [36,58]. EGFR activation by nontypeable *H*. *influenzae* attenuates host defensive and immune responses by negatively regulating the expression of Toll-like receptor 2 [37]. *K*. *pneumoniae* induces EGFR and its downstream signaling cascades to attenuate the activation of NF-κB, thereby modulating host immune responses [38]. *Pasteurella multocida* toxin exploits the transactivation of EGFR and subsequent mitogen-activated protein kinase activation to induce fibroblast proliferation [59]. In addition, *N*. *gonorrhoeae* modulates the activity and cellular distribution of host EGFR to facilitate its invasion and transmigration across the epithelium [33,34].

Meningitic *E*. *coli* strains have been shown to penetrate the BBB via a transcellular mechanism [2], but the underlying mechanisms remain incompletely understood. In this report, we demonstrate a novel role of EGFR in *E*. *coli* penetration of the BBB, a prerequisite for the development of *E*. *coli* meningitis. Noticeably, our chemical library screen using a model of *E*. *coli* invasion of HBMEC identified gefitinib, a selective inhibitor of EGFR tyrosine kinase, as an effective inhibitor of *E*. *coli* invasion of HBMEC. We, then, showed that EGFR contributed to *E*. *coli* penetration of the BBB, indicating that our approach of targeting *E*. *coli* invasion of HBMEC is likely to identify targets involved in the pathogenesis of *E*. *coli* meningitis.

Here, our demonstration of EGFR contribution to meningitic *E*. *coli* penetration of the BBB is provided by several lines of evidence. These include (a) pharmacological inhibition and knock-out of EGFR inhibited meningitic *E*. *coli* strain RS218 invasion of HBMEC *in vitro*, (b) administration of gefitinib effectively inhibited the penetration of circulating *E*. *coli* into the brain of neonatal mice, and (c) the co-localization of EGFR with the *E*. *coli* strain in HBMEC. These findings support the novel concept that EGFR is a biologically relevant host factor affecting *E*. *coli* penetration of the BBB, but the underlying mechanisms remain unclear.

We have previously shown that meningitic *E*. *coli* exploits specific host factors for invasion of the BBB, and that host factors contribute to invasion of the BBB by functioning as receptors for specific microbial factors and/or exploiting specific host cell signaling molecules [1–4]. If EGFR functions as a cell surface receptor for specific microbial factors, then blockade of EGFR would have affected *E*. *coli* binding to and invasion of the BBB. However, we found that pharmacological inhibition of EGFR blocked *E*. *coli* invasion of HBMEC in a dose-dependent manner and there was no effect on bacterial adhesion, indicating that EGFR is likely to contribute to *E*. *coli* invasion of the BBB by affecting host cell signaling molecules. This concept was also supported by our demonstration that three bacterial determinants contributing to *E*. *coli* invasion of the BBB (OmpA, FimH, NlpI) were involved in EGFR activation. Since these *E*. *coli* proteins were shown to interact with different host receptors [2,23–25], it is unlikely that EGFR functions as a co-receptor for different microbial-host factors. Studies are needed to elucidate how different microbial-host interactions exploit EGFR activation for *E*. *coli* penetration of the BBB.

S1P is recognized as a novel bioactive lipid mediator involved in physiological and pathogenic vascular functions [60]. For example, S1P regulates angiogenesis by activating the S1P_1_ and S1P_3_ receptors (GPCRs) on endothelial cells, which is required for endothelial cell morphogenesis into capillary-like networks [61]. S1P was also shown to induce adherens junction assembly through the G_i_/MAPK pathway and the small GTPase Rho and Rac pathways [61]. The S1P receptor S1P_1_ was shown to be essential for vascular maturation, and S1P_1_-mediated migration was defective in S1P_1_ mutant cells through their inability to activate the small GTPase Rac [62]. In addition, the S1P receptor S1P_2_ regulates vascular inflammation and atherosclerosis by inducing the release of inflammatory cytokines IL-1β and IL-18 and retaining macrophages in plaques [63]. Since various lines of evidence have shown that S1P functions in the vasculature and vascular-related cells [41,60], we investigated whether S1P is involved in microbial penetration of the BBB. Our data demonstrate for the first time a novel role of S1P and a potential crosstalk between EGFR and SphK2-S1P-S1P_2_ signaling in HBMEC upon meningitic *E*. *coli* infection. This novel concept was shown by our demonstrations (a) that S1P levels were significantly increased in HBMEC in response to meningitic *E*. *coli* infection, (b) that SphK2 inhibitors, but not SphK1 inhibitors, and S1P_2_ antagonist, but not S1P_1/3_ antagonist, inhibited *E*. *coli* invasion of HBMEC and (c) that the *E*. *coli* factors contributing to EGFR activation (OmpA, FimH, NlpI) were also shown to be involved in the activation of SphK2, and subsequent S1P generation in response to *E*. *coli* RS218. Moreover, S1P was shown to be an upstream signaling molecule of EGFR, by demonstrations that blockade of S1P function inhibited EGFR activation in response to meningitic *E*. *coli*, while blockade of EGFR function did not affect SphK2 activation. These findings prompted us to investigate how EGFR activation occurs, and to determine the role of S1P signaling in EGFR transactivation in meningitic *E*. *coli* invasion of the BBB.

To address these questions, we investigated the mechanisms of EGFR activation in response to meningitic *E*. *coli* in HBMEC. Activation of EGFR can occur upon binding of a specific ligand to its extracellular ligand-binding domain, leading to EGFR homo- and hetero-dimerization, and tyrosine phosphorylation of the cytoplasmic tyrosine kinase domain [31,51,52], and is classified as a ligand-dependent transactivation. A previous study on *N*. *gonorrhoeae* invasion of genital epithelial cells showed that EGFR transactivation occurred through up-regulation of several ligands including HB-EGF, AREG, and TGFα [33]. Here, we examined whether EGFR activation occurred in response to meningitic *E*. *coli* in a ligand-dependent manner by analyzing the expression levels of EGFR-related ligands. Our quantitative real-time PCR analysis revealed that upregulation of HB-EGF differed between HBMEC infected with wild-type meningitic *E*. *coli* and those infected with its triple mutant deleted of *ompA*, *fimH* and *nlpI*. Additionally, we were able to detect significant levels of HB-EGF in the supernatants of HBMEC infected with meningitic *E*. *coli* strain RS218, but not with the triple deletion mutant, suggesting that HB-EGF is likely to be involved in the activation of EGFR. In addition, HB-EGF is shown to bind to EGFR and ErbB4 [64], and our experiments here with EGFR knock-out and dominant-negative construct supported the involvement of EGFR in *E*. *coli* invasion of HBMEC. The contribution of HB-EGF was also shown by our demonstration that blockade of HB-EGF with CRM197 [55], which prevents ectodomain shedding of proHB-EGF, significantly inhibited EGFR activation as well as *E*. *coli* invasion of HBMEC. These findings support that the ligand-dependent action involving OmpA/FimH/NlpI exploitation of HB-EGF is likely to contribute to transactivation of EGFR in meningitic *E*. *coli* invasion of HBMEC.

Our time-dependent mRNA up-regulation of HB-EGF, however, did not follow the same time-dependent kinetics as EGFR activation. We showed that EGFR activation occurred in HBMEC as early as 15 min after infection with meningitic *E*. *coli*, while our real-time PCR analysis revealed that the mRNA level of HB-EGF was up-regulated at 60 min after infection, implying that EGFR activation in response to meningitic *E*. *coli* may occur via two mechanisms, comprising an early activation (perhaps representing a ligand-independent activation) and a late ligand-dependent activation involving HB-EGF. Previous studies have reported the crosstalk between GPCRs and EGFR, in which both ligand-independent and ligand-dependent activation of EGFR by GPCRs was demonstrated [65]. Here, our data showed that SphK2-S1P-S1P_2_ was upstream of EGFR in meningitic *E*. *coli* invasion, and more importantly that the EGFR activation at 30 min after infection was abolished by treatment with the S1P_2_ antagonist JTE-013, implying that the early EGFR activation might arise from the S1P-S1P_2_ pathway, which involves GPCR-related signaling. While S1P signaling occurred, it also trigged the late ligand-dependent activation of EGFR by regulating the expression of the EGFR-associated ligand HB-EGF. This is supported by the demonstration that blockade of S1P function with the SphK2 inhibitor and the S1P_2_ antagonist effectively prevented HB-EGF mRNA up-regulation at 60 min.

The ligand-dependent transactivation of EGFR by GPCRs is shown to occur by proteolytic processing of the transmembrane pro-EGFR-ligand precursor followed by paracrine activation of EGFR [53–55,65]. This process requires the participation of metalloproteinase activity, the so-called “Triple Membrane Passing Signal” (TMPS) mechanism. Therein, GPCRs stimulation induces metalloproteinase activity that cleaves the EGF-like ligand precursor and allows shedding of the ligand to bind to the extracellular ligand-binding domain of the receptor, thus transactivating EGFR signaling [65]. Therefore, the metalloproteinase-mediated shedding of EGF-like ligands might serve as a key step in GPCR-induced EGFR transactivation. In the present study, our finding for SphK2-S1P-S1P_2_ signaling to EGFR via HB-EGF was compatible with this TMPS mechanism. Noticeably, our results supported the exclusive involvement of HB-EGF, rather than other ligands, in EGFR activation in response to meningitic *E*. *coli*, and that is the reason for using CRM197 to specifically block HB-EGF function, rather than using a broad inhibitor of metalloproteinases. Nevertheless, several questions concerning the TMPS mechanism remain to be clarified for complete elucidation of the mechanisms underlying EGFR activation in response to meningitic *E*. *coli* invasion, such as identification of the specific member in the metalloproteinase family, the functional domain of metalloproteinases required for ligand precursor cleavage, and the manner in which activation occurs through GPCR signaling in HBMEC upon infection. At the present time, our findings support the involvement of S1P signaling in both early (ligand-independent) and late (ligand-dependent) activation of EGFR in response to meningitic *E*. *coli*. Further studies are needed to elucidate the mechanisms involved in the S1P-mediated early activation of EGFR in response to meningitic *E*. *coli* invasion of the BBB.

Meningitic *E*. *coli* triggers the activation of multiple host cell signal transduction pathways for invasion of the BBB [1–4]. The signaling molecules such as FAK and its associated cytoskeletal protein paxillin, phosphatidylinosital 3-kinase (PI3K), RhoGTPases, cytosolic phospholipase A2 (cPLA_2_) and ERM (ezrin, radixin, and moesin) protein family have all been identified to be involved in this process, mostly likely through their promoting actin cytoskeleton rearrangements in HBMEC [11,13,14,23,25–27,66,67]. Tyrosine-phosphorylated Src is found to be associated with EGFR upon stimulation of certain GPCRs. It has been shown to function as a mediator of the EGFR signaling pathway and can also be recruited by the activated EGFR phosphotyrosine domain [65]. The implication of host cell Src family tyrosine kinase in bacterial internalization has been reported in several pathogens. For example, ErbB2 phosphorylation results in recruitment and activation of the tyrosine kinase Src, which is dependent on the intrinsic kinase activity of ErbB2, and selective inhibitors of both ErbB2 and Src inhibited *N*. *meningitidis* internalization [35]. In Opa_52_-mediated phagocytosis of *N*. *gonorrhoeae* by human neutrophils, the activity of the Rho-family member Rac was controlled by a Src-like tyrosine kinase for efficient uptake [68]. Likewise, Src, together with Rho family GTPases, are involved in the internalization of *Shigella* into epithelial cells [69,70], and in triggering the formation of actin polymerization foci induced by *Shigella* [71]. Previously, c-Src tyrosine kinase was shown to regulate host cell actin cytoskeleton rearrangement and PI3K activation in HBMEC by *E*. *coli*, and contributed to *E*. *coli* invasion of HBMEC [57]. Here, we showed an association of c-Src with EGFR by co-immunoprecipitation analysis, and demonstrated that c-Src activation followed the SphK2-S1P-S1P_2_-EGFR signaling cascade and played an important role in meningitic *E*. *coli* invasion of the BBB. Additional studies are needed to elucidate the mechanisms involved with EGFR-c-Src signaling in *E*. *coli* invasion of the BBB.

**Fig 6:**
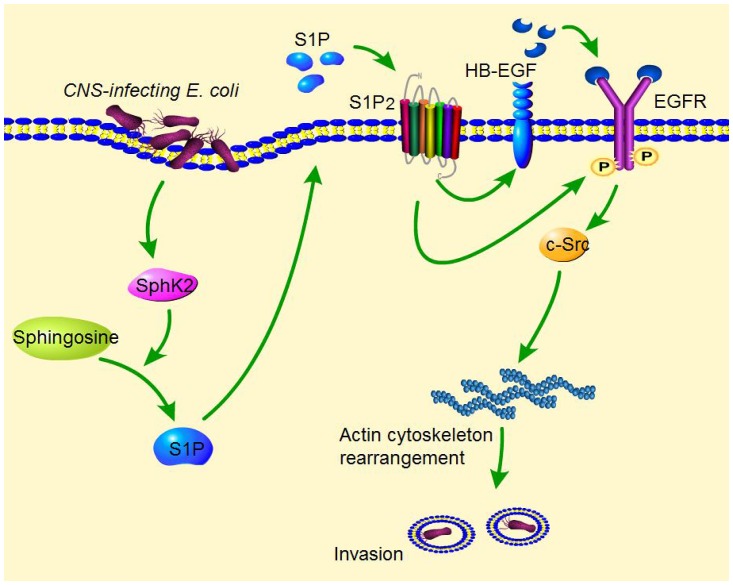
Schematic representation of the S1P-EGFR signaling pathway in meningitic *E*. *coli* invasion of HBMEC. Meningitic *E*. *coli* penetration of the BBB follows the microbial-host interactions contributing to HBMEC invasion, via exploiting specific host cell signaling molecules. During the HBMEC invasion, meningitic *E*. *coli* strains activate SphK2, which catalyzes the synthesis of S1P from sphingosine. S1P is then secreted outside and acts on S1P receptor S1P_2_. S1P interaction with S1P_2_ is involved in the activation of EGFR, as well as the up-regulation and release of EGFR-related ligand HB-EGF, which is proteolytically processed by metalloproteinases. The released HB-EGF binds to the extracellular ligand-binding domain of EGFR and leads to tyrosine phosphorylation of the EGFR cytoplasmic kinase domain. This SphK2-S1P-S1P_2_-EGFR cascade induces the activation of c-Src tyrosine kinase, an intracellular mediator that has been shown to regulate host cell actin cytoskeleton rearrangements, leading to *E*. *coli* invasion of HBMEC.

In summary, our findings report a novel mechanism exploited by meningitic *E*. *coli* for penetration of the BBB. Via its OmpA, FimH, and NlpI proteins, meningitic *E*. *coli* induces activation of SphK2, leading to increased generation of S1P that interacts with its receptor S1P_2_. This SphK2-S1P-S1P_2_ signaling is involved in early activation of EGFR, and also induces up-regulation and release of the EGFR-related ligand HB-EGF, which is responsible for transactivation of EGFR. Activated EGFR subsequently recruits c-Src and induces tyrosine phosphorylation of c-Src kinase, which promotes reorganization of the actin cytoskeleton in HBMEC and *E*. *coli* entry of the BBB (Fig 6). Hijacking of this SphK2-S1P-S1P_2_-EGFR-c-Src signaling cascade will facilitate meningitic *E*. *coli* to penetrate the BBB, the essential step in the development of *E*. *coli* meningitis. Recent reports have indicated that antimicrobial resistance is an emerging problem in *E*. *coli* causing meningitis [3,4], necessitating the development of novel targets for effective therapy. To our knowledge, this is the first demonstration that meningitic *E*. *coli* exploits S1P activation of EGFR for penetration of the BBB *in vivo* and *in vitro*, suggesting that S1P-EGFR represents a novel target for therapeutic development of *E*. *coli* meningitis.

## Materials and Methods

### Bacterial strains and cell culture

*E*. *coli* strain RS218 (O18:K1:H7) was obtained from cerebrospinal fluid of a neonate with meningitis [15]. All the mutants used in this study were derived from strain RS218 as previously described [9–11,15,20–22]. *E*. *coli* strains were cultured at 37°C overnight in brain heart infusion broth with appropriate antibiotics unless otherwise specified.

HBMEC were isolated and characterized as described previously [7]. Cells were routinely grown in RPMI 1640 containing 10% heat-inactivated fetal bovine serum, 10% Nu-Serum, 2 mM L-glutamine, 1 mM Sodium pyruvate, nonessential amino acids, vitamins, and penicillin and streptomycin (100 U/ml) in 37°C incubator under 5% CO_2_ until they reached confluence. In some experiments, confluent HBMEC were washed thrice with Hanks’ Balanced Salt Solution (Corning Cellgro, Manassas, VA, USA) and starved in serum-free medium (1:1 mixture of Ham’s F-12 and M-199) for 16–18 h before further treatment.

### Reagents, antibodies and plasmids

The EGFR tyrosine kinase inhibitor gefitinib, S1P receptors antagonists VPC23019 and JTE-013, and c-Src kinase inhibitor PP2 were purchased from Cayman Chemical Company (Ann Arbor, MI, USA). Other inhibitors of EGFR and ErbB were purchased from Selleck (Houston, TX, USA). The inhibitors of SphK1 and SphK2 and inactive analogue, including (*S*)-FTY720-vinylphosphonae, (*R*)-FTY720-methyl ether, RB-032, RB-033, and RB-034, were described previously [46–50] and used at 10 μM. The diphtheria toxin mutant CRM197 was obtained from BioAcademia Inc. (Osaka, Japan). Protein G Agarose Fast Flow beads and anti-phosphotyrosine (4G10) horseradish peroxidase (HRP)-conjugate antibody (used at 1:1000 for EGFR and c-Src phosphorylatyion detection) were purchased from EMD Millipore Corporation (Temecula, CA, USA). Anti-EGFR, anti-c-Src, and anti-c-Src-HRP conjugated antibodies were from Santa Cruz Biotechnology (1:1000) (Santa Cruz, CA, USA). A sphingosine kinase activation antibody kit was obtained from ECM Biosciences (Versailles, KY, USA). Cy3-conjugated antibody were purchased from Abcam (Cambridge, MA, USA). Anti-rabbit IgG HRP-conjugate antibody and Alexa Fluor 488-conjugated antibody were purchased from Cell Signaling Technology (Danvers, MA, USA). Anti-actin antibody was from Sigma-Aldrich (1:3000) (St. Louis, MO, USA). Preparations of OmpA-, FimH-, and NlpI-specific antibodies were previously described [20–22]. The TurboFect transfection reagent was purchased from Thermo Scientific (Suwanee, GA, USA) and used according to the instructions. G418 sulfate solution was from Corning Cellgro. The EGFR and c-Src dominant-negative constructs, pcDNA-EGFR-GGS and pEGFP-N1-Src-DN, along with their vector controls pcDNA3.1 and pEGFP-N1 were obtained from Drs. Hristova and Taylor, respectively [39,72].

### Chemical library screening

We used the Johns Hopkins Drug Library (JHDL), which is comprised of 3,400 chemicals that are approved by the US Food and Drug Administration and entered phase 2 clinical trials or approved for use abroad [73], for discovery of novel targets affecting *E*. *coli* invasion of HBMEC, as follows. Drugs were arrayed in 96-well plates and screened at a final concentration of 10 μM in DMSO (solvent). HBMEC grown in 96-well tissue culture plates were incubated with the JHDL for 60 min at room temperature, and then, examined for *E*. *coli* invasion, by a modification of the HBMEC invasion assay [9,10,15]. Briefly, 10 μl containing approximately 1×10^6^ CFUs of *E*. *coli* strain RS218 were inoculated into each well of HBMEC in the plate. The plates were incubated at 37°C for 90 min for bacterial invasion to occur, and then intracellular CFUs were determined. This screening assay always included *E*. *coli* strain RS218 in vehicle (DMSO)-treated HBMEC as a positive control for invasion, while bacteria without HBMEC were used as a control for assessing any inhibitory effect of the chemicals on the growth of *E*. *coli*. Since this JHDL contains antibiotics, those wells exposed to antibiotics were used as a positive control for identification of chemicals that inhibit *E*. *coli* growth. The assay was highly reproducible, and the coefficient of correlation from at least two separate experiments was r = 0.98 (*p*<0.0001). From this assay, we identified gefitinib, which inhibited meningitic *E*. *coli* invasion of HBMEC greater than 90%. It is important to note that gefitinib did not affect bacterial growth, as assessed by comparing CFUs in experimental medium with or without the drug and also did not affect HBMEC viability, as assessed by live/dead stain (Molecular Probes). It is also important to note that EGFR has not been previously appreciated to affect *E*. *coli* penetration of the BBB.

### Bacterial adhesion and invasion assays in HBMEC

The ability of *E*. *coli* to bind to and invade HBMEC was determined as previously described [9–12,15,20–22]. Briefly, *E*. *coli* strains were grown overnight in brain heart infusion broth with streptomycin (50 μg/ml). Bacteria were resuspended in experimental medium (M199-Ham F12 [1:1] medium containing 5% heat-inactivated FBS) and added into the confluent HBMEC monolayer grown in 24-well plate at MOI of 100. The plate was incubated at 37°C incubator with 5% CO_2_ for 90 min to allow binding. HBMECs were then washed three times to remove unbound bacteria, and lysed in 0.025% Triton X-100 buffer. Bacterial counts of adhesion were determined by plating with appropriate dilutions. For invasion assay, bacteria were added into HBMEC as described above for the adhesion assay. Subsequently, cells were washed three times to remove the unbound bacteria and incubated in experimental medium containing 100 μg/ml gentamicin for another 1 h to kill extracellular bacteria. HBMEC were washed and lysed as above mentioned. The released intracellular bacteria were quantified by appropriate dilutions and plating. As specified in some experiments, HBMEC were pretreated with various inhibitors for 1 h prior to addition of bacteria and then processed for bacterial invasion. The results were calculated as percentages of the initial inoculums, and presented as percent relative adhesion/invasion compared with that in the presence of the vehicle control (DMSO). Each assay was performed in triplicate.

### MTT assay

MTT Cell Proliferation Assay Kit was purchased from BioVision (Milpitas, CA, USA) and used according to the instructions. HBMEC were seeded in 96 well plates at 5×10^3^ per well in 100 μL culture medium and incubated for 24 h. Gefitinib was added as indicated in HBMEC binding and invasion assays. Supernatant of each well was removed and MTT dissolved in serum-free medium was added and further incubated for another 4 h. After incubation, 100 μL of MTT solvent was added into each well, and the plate was wrapped in a foil and shaked on an orbital shaker for 15 min. Absorbance of all wells at 570 nm were determined.

### Mass spectrometry analysis of the sphingolipids in HBMEC

HBMEC monolayers grown in 100-mm dish were serum-starved overnight and incubated with *E*. *coli* strain RS218 or the triple deletion mutant at a MOI of 100 for 30 min at 37°C. Sphingolipids were extracted using acidified organic solvents and quantitated by HPLC electrospray ionization triple quadrupole mass spectrometry and quantitated using mass labeled internal standards [74]. Briefly, sphingolipids were extracted from cell lysates as previously described [74,75]. Prior to extraction, a mixture of C17 sphingolipids (125 pmol/sample) was added to each sample as the internal standards. Sphingolipids were quantitated by HPLC electrospray ionization tandem mass spectrometry using selected ion monitoring on an ABSciex 4000 Q-Trap instrument as described previously [76–78]. Total phospholipids for each sample were measured using modified Ames and Dubin assay as previously described [75,79].

### Transfection

HBMEC were transfected with empty vectors control or expression vectors encoding EGFR or c-Src dominant-negative constructs using the TurboFect transfection reagent as described previously [66,80]. pcDNA3.1 cloned with green fluorescence protein (GFP) and pEGFP-N1 vectors were used to determine the transfection efficiency by fluorescence microscopy.

### Genome editing via CRISPR-Cas9

A human codon-optimized Cas9 expression vector was obtained from Addgene, plasmid #41815 [81], and Cas9 was cloned into the pEF6 expression vector (Invitrogen, Carlsbad, CA, USA), downstream and in-frame with a nuclear-localized YFP, linked by a piconaviral 2A bicistronic peptide [82], such that nuclear localization signal (NLS)-YFP and Cas9 are expressed in approximate equimolar quantities. A hEGFR guide RNA (gRNA) construct, including the U6 promoter, was synthesized as a double stranded DNA fragment and cloned into the pEF6-nls-YFP-2A-Cas9 vector by InFusion Cloning (Clontech, Mountain View, CA, USA). This vector was used for transfection of HBMEC as mentioned above and clones resistant to blasticidin were identified and used for isolation of a single clone. Single clones were used for expression of EGFR by Western blot and bacterial invasion assay.

### Immunoprecipitation and western blotting

HBMEC were seeded at 1×10^6^ cells/100-mm dish and cultured until confluence. Cells were then serum-starved overnight, stimulated with *E*. *coli* strains at MOI of 100 for specified periods of time, and processed for immunoprecipitation and Western blotting analysis as previously described [66,80].

### RNA isolation and real-time PCR

Confluent HBMEC grown in 100-mm dishes were serum-starved overnight and then infected with *E*. *coli* at a MOI of 100 for indicated periods of time. At each time point, the medium was removed and the cells were lyzed for total RNA preparation using the TRIzol reagent (Invitrogen). Contaminating DNA was removed by DNase I treatment (New England Biolabs, Ipswich, MA, USA). Aliquots (1 μg) of the total RNA in each sample were subjected to cDNA synthesis using ProtoScript Taq RT-PCR kit (New England Biolabs). Real-time PCR was performed with a QuantStudio 12K Flex Real-Time PCR System (Applied BioSystems, Foster City, CA, USA) using Power SYBR Green PCR master mix (Applied BioSystems), according to the manufacturers’ instructions. The primer sequences for human EGFR and its ligands were as follows: EGFR, 5'-CAAGTGCTGGATGATAGA-3' (forward) and 5'-GAAGTTGGAGTCTGTAGG-3' (reverse); EGF, 5'-GTTGGCAGGTGGTGAAGTTG-3' (forward) and 5'-CCACAGGAGCACAGTCATCT-3' (reverse); AREG, 5'-ATTATGCTGCTGGATTGG-3' (forward) and 5'-GAGGACGGTTCACTACTA-3' (reverse); BTC, 5'-CCAAGCAATACAAGCATTAC-3' (forward) and 5'-GTCCTCTGTCTCCTCTTAG-3' (reverse); EREG, 5'-AGTTCAGACAGAAGACAATC-3' (forward) and 5'-ACATCGGACACCAGTATA-3' (reverse); HB-EGF, 5'-TATACCTATGACCACACAAC-3' (forward) and 5'-CACATCATAACCTCCTCTC-3' (reverse); TGFα, 5'-GGCTGTCCTTATCATCAC-3' (forward) and 5'-AGACCACTGTTTCTGAGT-3' (reverse). Primers for human glyceraldehyde-3-phosphate dehydrogenase (GAPDH) were provided in the RT-PCR kit. The amplification conditions were: 50°C for 2 min and 95°C for 10 min, followed by 40 cycles of 95°C for 15 s and 60°C for 1 min. The products were then applied to a melt curve stage with denaturation at 95°C for 15 s, anneal at 60°C for 1 min, and slow dissociation by ramping from 60°C to 95°C at 0.05°C/s to ensure the specificity of the PCR products.

### Determination of secretory HB-EGF from HBMEC by ELISA

To determine the release of secretory HB-EGF from HBMEC, cells were infected with meningitic *E*. *coli* strain RS218 or its triple deletion mutant for varying time points, and the supernatants were discarded and cells were washed with 1.5 M NaCl/1×PBS/1% BSA to dissolve heparin-bound HB-EGF. Cleaved and secretory HB-EGF levels were then quantified from wash buffer using HB-EGF Human ELISA Kit, purchased from Abcam (Cambridge, MA, USA), according to the manufacturer’s instructions.

### Animal infection assay

C57BL/6j mice were purchased from Jackson Laboratory (Bar Harbor, Maine). SphK2^−/−^ mice in the background of C57BL/6 were described previously [42]. Male or female mice at 1 week of age were used for induction of hematogenous *E*. *coli* meningitis. All procedures and handling techniques were approved by the Animal Care and Use Committee of the Johns Hopkins University. Each mouse received approximately 3×10^5^ CFU of *E*. *coli* strain RS218 in 50 μl sterile normal saline via intracardiac injection. At 1 h post-inoculation, the mice were euthanized and blood from the right ventricle was collected for quantitative bacterial cultures. Subsequently, the mice were perfused as previously described [14], and their brains were removed, weighed, homogenized, and plated to determine the bacterial counts, which were expressed as CFUs per gram. In some experiments, gefitinib, applied at therapeutic dosage (10 mg/kg) [83], was intraperitoneally administrated 2 h before bacterial challenge.

### Immunofluorescence of HBMEC

HBMEC was grown on collagen-coated glass slide to confluency. Cells were washed thrice with serum-free medium and then pre-incubated for 30 minutes in experimental medium. Cells were then incubated with *E*. *coli* containing a red fluorescence protein (RFP)-expressing plasmid (RS218-RFP), at an MOI of 1:100 for a period of 90 minutes at 37°C with 5% CO_2_. Cells were washed with PBS to remove the free, unbound bacteria, and then fixed with 4% paraformaldehyde, permeabilized with Triton X-100 solution, and blocked with 5% BSA in PBS. Cells were then incubated with EGFR antibody overnight at 4°C, washed, and incubated with Alexa Fluor 488-labeled secondary antibody (Life Technologies A11034), followed by nucleus staining with DAPI (Vector Laboratories H-1200). The glass slide was mounted and visualized using fluorescence microscopy.

### Statistical analysis

Data were expressed as mean ± standard errors of the mean (SEM) unless otherwise noted. Differences of the bacterial counts in adhesion and invasion assays were determined by Student’s *t*-test. Differences of the bacterial counts between different treatments or groups of mice were determined by the Wilcoxon rank-sum test. *P*-values of < 0.05 were considered significant.

### Ethics statement

This study was carried out in strict accordance with the current recommendations in the Guide for the Care and Use of Handling Animals, NIH publication DHHS/USPHS. The animal protocol was approved by The Johns Hopkins Animal Care and Use Committee (Animal Welfare Assurance Number: A3272-01). All efforts were made to provide the ethical treatment and minimize suffering of animals (mice) employed in this study.
